# Supplementary material for: Adapting the Intensity Gradient for Use with Count-Based Accelerometry Data in Children and Adolescents
Source: Sensors (Basel). 2024 May 10;24(10):3019. doi: 10.3390/s24103019 (PMC11125286; doi:10.3390/s24103019)
Supplement: Supplementary file 1 [file sensors-24-03019-s001.zip › sensors-2935401-supplementary.pdf]

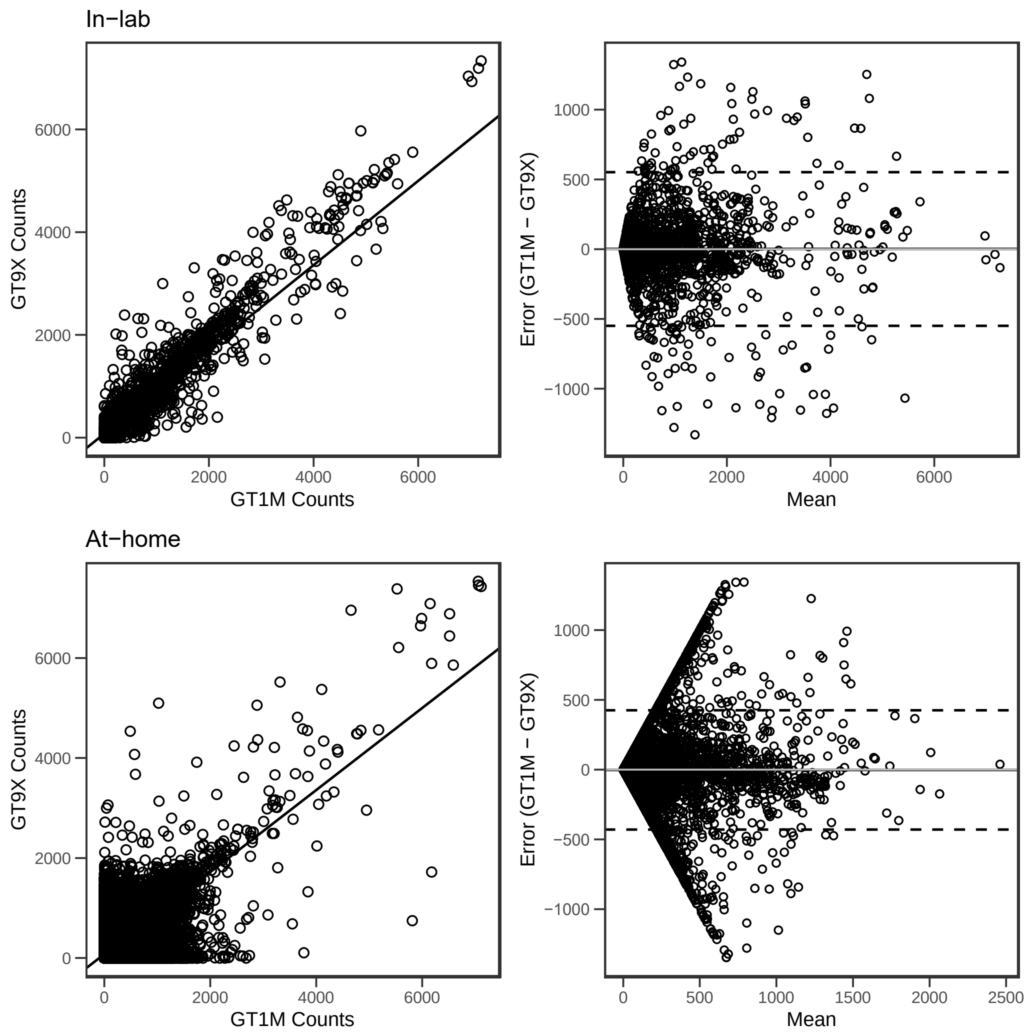

Figure S1: Linear regression and Bland-Altman plots for combined in-lab and at-home count data. Linear regressions show the line of best fit; the Bland-Altman plots show the mean difference as the solid black line, the zero line as the solid grey line, and upper and lower 95% limits of agreement as dotted lines.

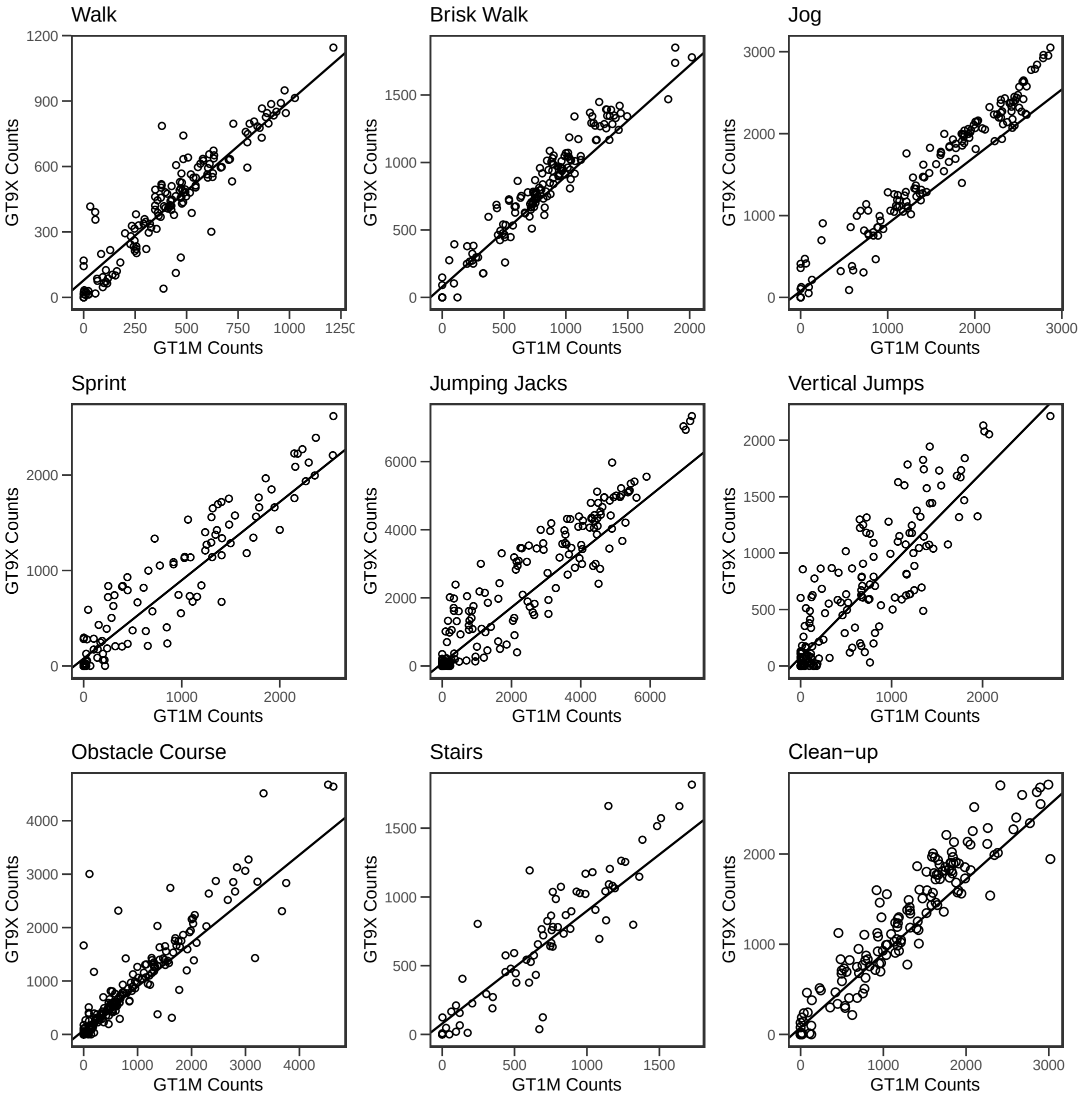

Figure S2: Linear regression plots for each individual activity performed during the in-lab testing session. Data from all participants are combined. Plots show the line of best fit based on multiple linear regression [17].
